# Supplementary material for: Underlying drivers of coral reef vulnerability to bleaching in the Mesoamerican Reef
Source: Commun Biol. 2024 Nov 6;7:1452. doi: 10.1038/s42003-024-07128-y (PMC11541557; doi:10.1038/s42003-024-07128-y)
Supplement: Supplementary file 1 — Supplementary information [file 42003_2024_7128_MOESM1_ESM.pdf]

Supplementary Material

Underlying drivers of coral reef vulnerability to bleaching in the Mesoamerican Reef

Aarón Israel Muñiz-Castillo[1, 2*], Andrea Rivera-Sosa[1], Melanie McField[2,3*], Iliana Chollett[4], C. Mark Eakin[5], Susana Enríquez[6], Ana Giró[2], Ian Drysdale[2], Marisol Rueda[2], Melina Soto[2], Nicole Craig[2], Jesús Ernesto Arias-González[1*]

[1] Laboratorio de Ecología de Ecosistemas de Arrecifes Coralinos, Departamento de Recursos del Mar, Centro de Investigación y de Estudios Avanzados del Instituto Politécnico Nacional. Mérida, Yucatán 97310, Mexico.
[2] Healthy Reefs for Healthy People, USA; Mexico; Guatemala; Belize; and Honduras.
[3] Smithsonian Marine Station, Smithsonian Institution, Fort Pierce 34949 Florida, USA.
[4] Sea Cottage, Louisburgh, Co. Mayo, Ireland.
[5] Corals and Climate, Silver Spring 20904, Maryland, USA.
[6] Laboratorio de Fotobiología. Unidad Académica de Sistemas Arrecifales Puerto Morelos, Instituto de Ciencias del Mar y Limnología, Universidad Nacional Autónoma de México, (UNAM), Apdo. Postal #13, Cancun, Quintana Roo 77500, Mexico.

*Corresponding authors: Aarón Israel Muñiz-Castillo, israel@healthyreefs.org; Melanie McField, mcfield@healthyreefs.org; Jesús Ernesto Arias-González, earias@cinvestav.mx

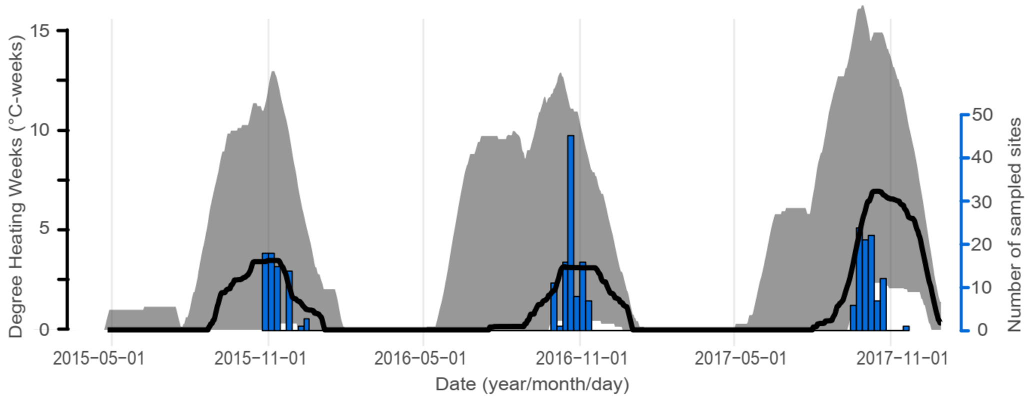

Supplementary Figure 1. Degree Heating Weeks (accumulated heat stress) and frequency of sampled sites in the sampling season. The black line represents the median DHWs observed on each of the days, and the shading represents the maximum and minimum DHWs on each of the days (considering 1,861 pixels per day for the entire MAR coral reef region). For the construction of this plot of temporal variation of DHWs, we used the information available at https://www.ncei.noaa.gov/archive/accession/0205786. The blue bars represent the number of sites sampled over the period 2015-2017.

Supplementary Table 1. Statistical descriptors of the Bleaching Severity Index (BSI), percentage of whole bleached colonies (BL = bleaching coverage > 90%), percentage of partially bleached colonies (PB = bleaching coverage of 0 - 90%), and percentage of pale colonies (P).

Year	Category	Min	1st Qu.	Median	Mean	3rd Qu.	Max	SD
	BSI	0.00	17.06	29.70	28.85	39.72	60.27	14.86
All years	BL	0.00	1.01	3.52	6.01	7.89	39.13	7.07
(n = 266)	PB	0.00	9.79	21.04	22.93	34.10	60.39	15.18
	P	0.00	15.22	23.23	22.66	29.16	52.00	10.21
	BSI	0.00	7.58	17.00	18.96	28.17	46.67	12.56
2015	BL	0.00	0.00	1.48	2.78	4.81	16.50	3.50
(n = 69)	PB	0.00	4.50	11.00	14.63	22.60	40.98	11.99
	P	0.00	10.45	15.17	19.27	26.62	52.00	12.17
	BSI	0.79	16.92	28.10	25.67	34.26	51.83	11.66
2016	BL	0.00	0.90	1.93	3.54	4.79	20.00	4.24
(n = 104)	PB	0.39	9.49	20.56	21.29	31.97	49.25	13.54
	P	0.39	16.27	25.74	23.80	30.15	48.34	10.63
	BSI	12.33	29.54	42.55	39.74	50.45	60.27	12.75
2017	BL	0.35	5.00	9.26	11.16	15.38	39.13	8.56
(n = 93)	PB	2.87	18.50	30.50	30.93	42.26	60.39	15.26
	P	2.83	19.14	24.00	23.89	28.50	42.00	7.24

Supplementary Table 2. Yuen's test (robust t-test) on trimmed means for dependent samples considering only re-sampled sites.

Years	n	Df	Category	Mean difference	CI lower	CI upper	test value	p-value	Explanatory measure of effect size
2015 - 2016	49	40	BSI	-2.43	-6.27	1.42	-1.28	0.21	0.13
2015 - 2016	49	40	BL	-1.23	-2.91	0.45	-1.48	0.15	0.23
2015 - 2016	49	40	PB	-0.37	-4.26	3.52	-0.19	0.85	0.02
2015 - 2016	49	40	P	-0.12	-3.83	3.60	-0.06	0.95	0.01
2015 - 2017	29	24	BSI	-13.31	-19.66	-6.96	-4.32	0.02	0.60
2015 - 2017	29	24	BL	-6.03	-9.30	-2.75	-3.79	0.09	0.75
2015 - 2017	29	24	PB	-8.61	-13.31	-3.91	-3.78	0.09	0.39
2015 - 2017	29	24	P	-0.72	-6.57	5.13	-0.25	0.80	0.04
2016 - 2017	67	54	BSI	-13.82	-16.26	-11.37	-11.32	6.6e-16	0.68
2016 - 2017	67	54	BL	-7.91	-9.90	-5.91	-7.96	1.17e-10	0.82
2016 - 2017	67	54	PB	-9.04	-12.83	-5.25	-4.78	1.0e-05	0.38
2016 - 2017	67	54	P	1.32	-1.56	4.20	0.92	0.36	0.12

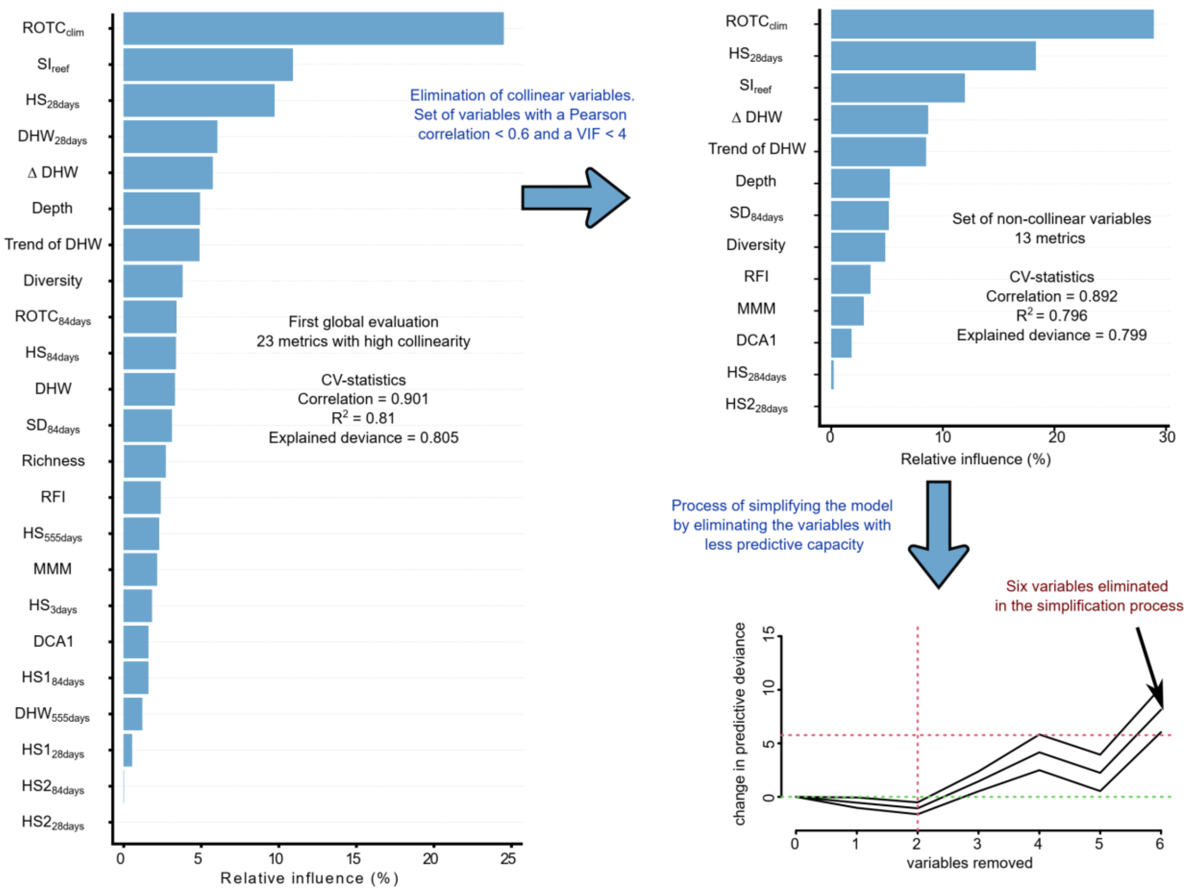

Supplementary Fig. 2. Variable selection process and development of the Gradient Boosted Model.

Supplementary Table 3. Friedman's H-statistic to assess the strength of the interactions. Scaled values from 0 (null interaction) to 1 (complete effect of interaction) with the strength of interactions in the model.

	ROTCclim	HS_{28days}	SI_{reef}	Δ DHW	Trend of DHW	Diversity
HS_{28days}	0.13	-	-	-	-	-
SI_{reef}	0.15	0.07	-	-	-	-
Δ DHW	0.22	0.03	0.14	-	-	-
Trend of DHW	0.16	0.12	0.02	0.04	-	-
Diversity	0.04	0.10	0.14	0.12	0.08	-
Depth	0.08	0.09	0.08	0.19	0.11	0.12

Supplementary Table 4. Bleaching severity of species considering all years (2015-2017). The species are ordered from the most to the least affected.

Specie	Bleaching severity	% bleaching	% partially bleached	% pale	N° of colonies
Millepora squarrosa	70.83	50.00	25.00	12.50	8
Solenastrea hyades	50.00	0.00	50.00	50.00	2
Millepora striata	44.67	10.00	42.00	20.00	50
Agaricia tenuifolia	41.98	5.02	43.35	24.17	6355
Orbicella franksi	41.75	10.18	38.13	18.45	737
Orbicella annularis	40.98	8.35	37.36	23.15	3209
Agaricia fragilis	40.00	14.17	30.00	17.50	120
Scolymia lacera	40.00	20.00	20.00	20.00	5
Porites furcata	39.40	10.30	33.94	19.45	1049
Hellioseris cucullata	37.20	9.07	37.11	10.20	353
Agaricia humilis	36.31	7.74	35.71	14.29	168
Agaricia agaricites	35.55	9.54	29.09	19.85	6455
Siderastrea siderea	34.89	9.46	23.54	29.19	7174
Porites porites	33.19	8.09	27.86	19.58	2053
Isophyllia rigida	32.42	10.96	21.92	20.55	73
Mussa angulosa	32.18	17.24	17.24	10.34	29
Agaricia lamarcki	31.73	4.81	32.69	15.38	104
Isophylia sinuosa	30.77	3.85	23.08	34.62	26
Orbicella faveolata	30.28	5.15	25.64	24.10	2465
Mycetophyllia lamarckia	29.47	9.44	15.45	29.18	233
Scolymia cubensis	29.41	17.65	5.88	23.53	17
Diploria labyrinthiformis	29.11	6.15	22.79	23.30	781
Mycetophyllia ferox	29.10	4.76	19.05	34.92	63
Acropora cervicornis	28.90	1.53	32.54	17.03	1180
Millepora complanata	27.28	8.25	20.93	15.23	1333
Colpophyllia natans	25.03	5.11	15.97	27.80	939
Porites divaricata	23.92	5.65	16.67	21.51	372
Solenastrea bournoni	23.91	4.35	21.74	15.22	92
Acropora prolifera	23.77	1.64	23.77	18.85	122
Pseudodiploria clivosa	23.56	3.91	9.12	40.72	307
Stephanocoenia intersepta	22.28	4.43	17.84	17.84	835
Millepora alcicornis	21.38	7.69	12.84	15.38	1612
Eusmilia fastigiata	20.75	2.07	17.84	20.33	241
Siderastrea radians	20.33	6.10	9.35	23.98	246
Porites astreoides	19.58	4.97	12.58	18.66	7931
Madracis auretenra	19.55	0.00	15.79	27.07	133
Pseudodiploria strigosa	19.50	1.71	8.55	36.26	2747
Dendrogyra cylindrus	19.43	1.23	17.18	20.25	163
Meandrina jacksoni	17.09	0.00	10.26	30.77	39
Manicina areolata	16.67	2.63	5.26	31.58	38
Madracis decactis	16.67	1.97	15.13	13.82	152
Dichocoenia stokesii	15.66	2.56	12.43	14.44	547
Montastraea cavernosa	14.00	1.23	8.15	22.03	2528
Favia fragum	13.16	7.89	5.26	5.26	38
Meandrina meandrites	12.65	0.96	8.67	17.73	519
Mycetophyllia aliciae	10.82	2.63	6.14	12.28	114
Acropora palmata	9.58	0.34	8.79	10.15	887

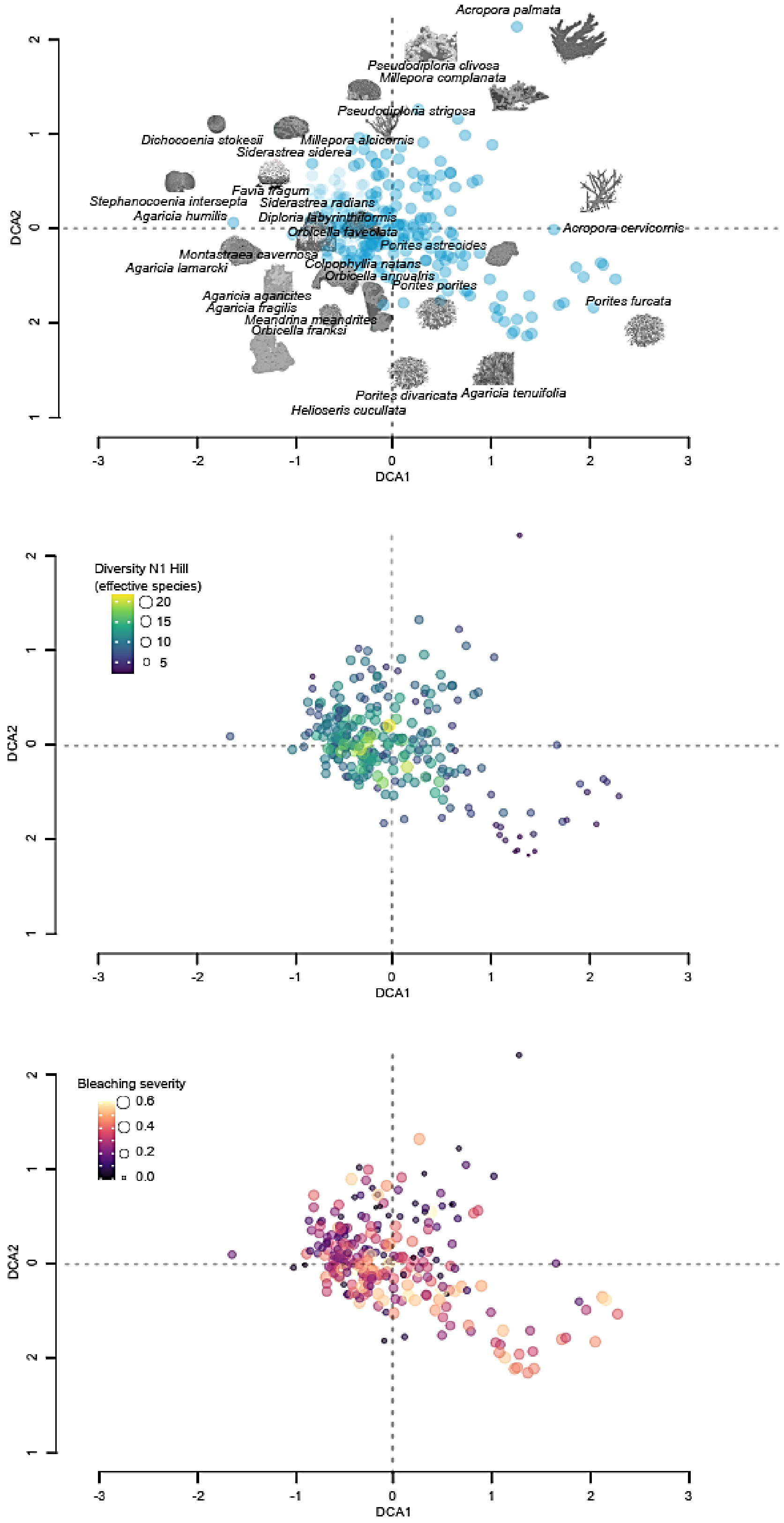

Supplementary Figure 3. Detrended correspondence analysis (DCA) ordination plot of the coral community. Gray points represent the site's ordination based on coral species composition. Only the 28 most abundant species are shown, with an image of the typical morphology of some coral species.

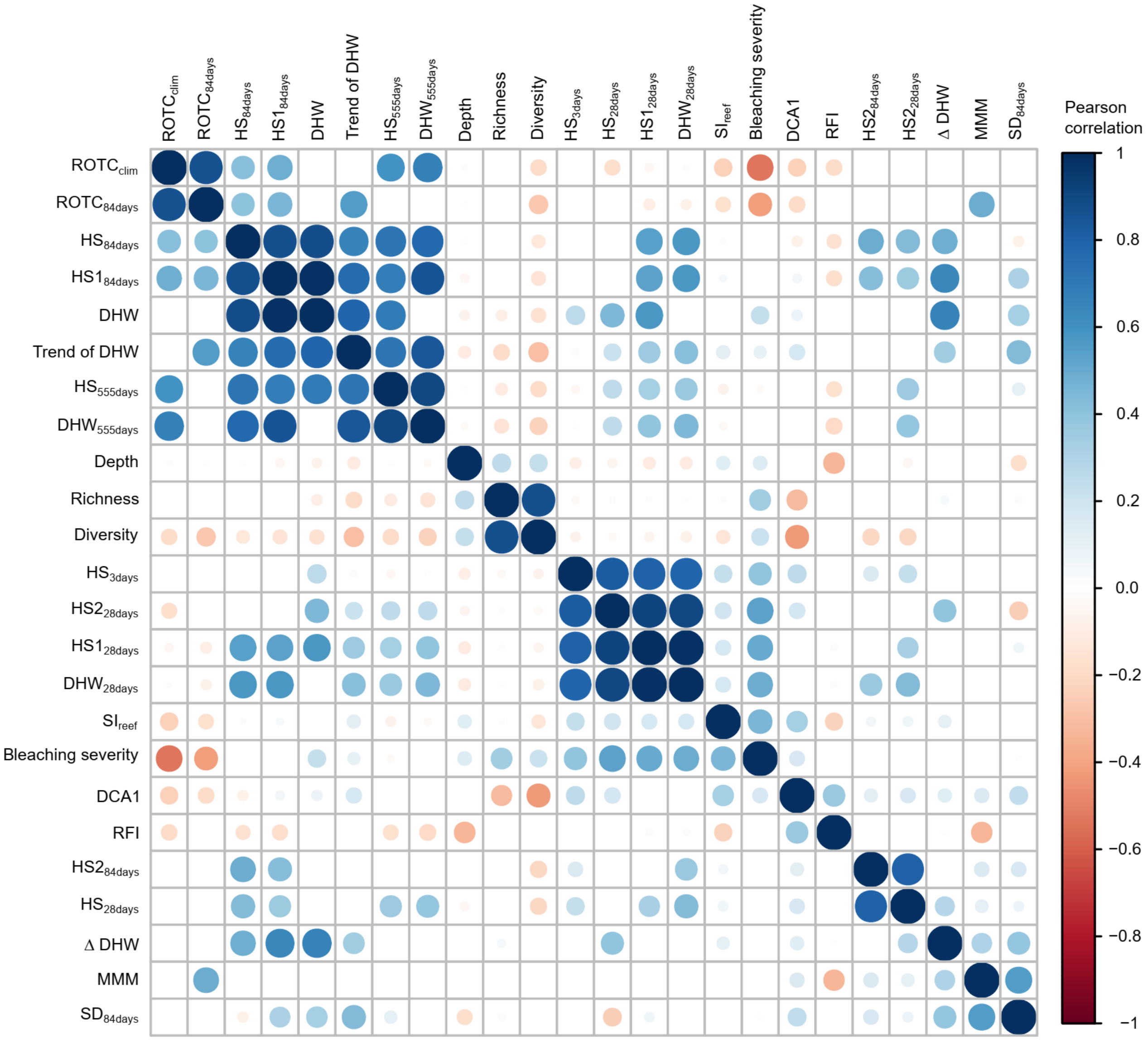

Supplementary Figure 4. Pearson's correlation between the variables considered in the analysis. The plot only shows significant correlations with a p-value < 0.05. Variables are explained below:

1. $ROTC_{clim}$: The seasonal warming rate (ROTC) reflects the trend in temperature change over 84 weeks during summer. The climatological seasonal-warming rate (ROTCclim) is the average of the annual ROTC values for the period 1985-2012 (*sensu* Chollett et al. 2014)[1].
2. $ROTC_{84days}$: Maximum ROTC observed 84 days before sampling date[1].
3. MMM: Monthly average of the hottest month registered during 1985-2012[2].
4. SD_{84days}: Standard deviations of the SST in the previous 84 days.
5. $HS2_{84days}$: Number of days with HS values greater than 2 °C in the last 84 days[3]
6. $HS2_{28days}$: Number of days with HS values greater than 2 °C in the last 28 days[3].
7. HS_{84days}: Sum of HS in 84 days before the sampling date[3].
8. $HS1_{84days}$: Number of days with HS values greater than 1 °C in the last 84 days[3].
9. DHW: Conventional DHW calculation considering an 84-day window[2].
10. Trend of DHW: DHW quantifies heat stress by summing up positive daily anomalies above 1 °C above the MMM over 84 days (12 weeks), divided by 7 to express values per week. 'Trend of DHW' is the trend of annual maximums DHWs from 1985 to the sampling year, a trend obtained from a generalized least square model[4].
11. $HS5_{55days}$: Sum of HS in 555 days before the sampling date.

12. $DHW_{555days}$: DHW calculation considering a 555-day window represents the accumulated stress since the beginning of the summer of the previous year.
13. Richness: Number of coral species.
14. Diversity: The diversity of corals calculated from Hill's number one equal to Shannon's diversity exponent, this represents true diversity without considering the less abundant or "rare" species[5].
15. Δ DHW: Difference between the maximum observed value of DHW in the current event up to the sampling date and the maximum observed value of DHW in the last year (building on Hughes 2019[6]).
16. Depth: Mean depth of the reef in meters.
17. DCA1: First axis in a multidimensional ordination analysis based on species composition and applying a Detrended Correspondence Analysis. Considered a potential ecological gradient related to bleaching[3]
18. RFI: Reef Functional Index based on the relative abundance of species multiplied by a functional coefficient[7].
19. HS_{3days}: Hotspots (HS) represent positive daily anomalies above the MMM[2]. HS_{3days} is the average HS in the last three days before the sampling date.
20. HS_{28days}: Sum of HS in 28 days before the sampling date.
21. $HS1_{28days}$: Number of days with HS values greater than 1 °C in the last 28 days[3].
22. DHW_{28days}: DHW calculation considering a 28-day window[3].
23. SI_{reef}: Reef sensitivity based on the relative abundance of species weighted by species response to bleaching[8].
24. Bleaching severity: The bleaching severity index (was adapted from the Bleaching and Mortality Indexes (BMI)[9] and calculated from the sum of the proportion of colonies in each response category, weighting each category according to its ecological impact.

Supplementary Table 5. Partner Institutions and Organizations Involved in Data Collection for the Coral Bleaching Study during 2015-2017 in the Mesoamerican Reef Region.

Country	Area	Partners	Healthy Reefs contact	Contact
Mexico	Puerto Morelos	Universidad Nacional Autónoma de México	Marisol Rueda and Melina Soto	rueda@healthyreefs.org; soto.melina@gmail.com
	Akumal	Centro Ecologico Akumal		
	Cozumel	Comisión Nacional de Áreas Naturales Protegidas		
	Tulum	Global Vision International / Amigos de Sian Ka'an		
	Xcalak	Comisión Nacional de Áreas Naturales Protegidas		
	Southern Quintana Roo	Oceanus		
Belize	Lighthouse Reef Atoll	Belize Audubon / Blue Ventures	Nicole Craig and Melanie McField	craig@healthyreefs.org; mcfield@healthyreefs.org
	Central and Southern Barrier	Fragments of Hope / Southern Environmental Association / Toledo Institute for Development and Environment / Belize Fisheries Department		
Guatemala	Coastal Guatemala	Consejo Nacional de Areas Protegidas / Fundación Para el Ecodesarrollo y la Conservación / Semillas del océano	Ana Giro	giro@healthyreefs.org
Honduras	Tela	Amigos de los Arrecifes de Tela / Centro de Investigación y de Estudios Avanzados del Instituto Politécnico Nacional	Ian Drysdale	drysdale@healthyreefs.org
	Roatan and Cayo Cochinos	Coral Reef Alliance / Centro de Investigación y de Estudios Avanzados del Instituto Politécnico Nacional / Center for Marine Ecology		
	Utila	Bay Islands Conservation Roatán / Coral Reef Alliance		

References

1. Chollett, I., Enríquez, S. & Mumby, P. J. Redefining Thermal Regimes to Design Reserves for Coral Reefs in the Face of Climate Change. *PLoS One* **9**, e110634 (2014).

2. Liu, G. *et al.* Reef-scale thermal stress monitoring of coral ecosystems: New 5-km global products from NOAA coral reef watch. *Remote Sens (Basel)* **6**, 11579–11606 (2014).

3. Safaie, A. *et al.* High frequency temperature variability reduces the risk of coral bleaching. *Nat Commun* **9**, 1671 (2018).

4. Muñiz-Castillo, A. I. *et al.* Three decades of heat stress exposure in Caribbean coral reefs: a new regional delineation to enhance conservation. *Sci Rep* **9**, 11013 (2019).

5. Jost, L. Entropy and diversity. *Oikos* **113**, 363–375 (2006).

6. Hughes, T. P. et al. Ecological memory modifies the cumulative impact of recurrent climate extremes. Nat. Clim. Chang. 9, 40–43 (2019).

7.González-Barrios, F. J. & Álvarez-Filip, L. A framework for measuring coral species-specific contribution to reef functioning in the Caribbean. *Ecol Indic* **95**, 877–886 (2018).

8. McClanahan, T. R. *et al.* Temperature patterns and mechanisms influencing coral bleaching during the 2016 El Niño. *Nat Clim Chang* **9**, 845–851 (2019).

9. McClanahan, T. R. The relationship between bleaching and mortality of common corals. *Mar Biol* **144**, 1239–1245 (2004).
