# Supplementary material for: Underlying drivers of coral reef vulnerability to bleaching in the Mesoamerican Reef
Source: Commun Biol. 2024 Nov 6;7:1452. doi: 10.1038/s42003-024-07128-y (PMC11541557; doi:10.1038/s42003-024-07128-y)
Supplement: Supplementary file 2 — Description of Additional Supplementary Files [file 42003_2024_7128_MOESM2_ESM.pdf]

Description of Additional Supplementary Files

File name: Supplementary Data 1

Description: Spatiotemporal Variation of Coral Bleaching in the Mesoamerican Reef (2015-2017)

File name: Supplementary Data 2
Description: Site-Specific Predictors of Coral Bleaching Severity in the Mesoamerican Reef (2015-2017)
