# Supplementary material for: Underlying drivers of coral reef vulnerability to bleaching in the Mesoamerican Reef
Source: Commun Biol. 2024 Nov 6;7:1452. doi: 10.1038/s42003-024-07128-y (PMC11541557; doi:10.1038/s42003-024-07128-y)
Supplement: Supplementary file 5 — Reporting summary [file 42003_2024_7128_MOESM5_ESM.pdf]

nature portfolio

Corresponding author(s): A. Israel Muñiz Castillo

Last updated by author(s): Oct 9, 2024

Reporting Summary

Nature Portfolio wishes to improve the reproducibility of the work that we publish. This form provides structure for consistency and transparency in reporting. For further information on Nature Portfolio policies, see our Editorial Policies and the Editorial Policy Checklist.

Statistics

For all statistical analyses, confirm that the following items are present in the figure legend, table legend, main text, or Methods section.

n/a	Confirmed	
☐	☒	The exact sample size (n) for each experimental group/condition, given as a discrete number and unit of measurement
☐	☒	A statement on whether measurements were taken from distinct samples or whether the same sample was measured repeatedly
☐	☒	The statistical test(s) used AND whether they are one- or two-sided<br>*Only common tests should be described solely by name; describe more complex techniques in the Methods section.*
☐	☒	A description of all covariates tested
☐	☒	A description of any assumptions or corrections, such as tests of normality and adjustment for multiple comparisons
☐	☒	A full description of the statistical parameters including central tendency (e.g. means) or other basic estimates (e.g. regression coefficient) AND variation (e.g. standard deviation) or associated estimates of uncertainty (e.g. confidence intervals)
☐	☒	For null hypothesis testing, the test statistic (e.g. F, t, r) with confidence intervals, effect sizes, degrees of freedom and P value noted<br>*Give P values as exact values whenever suitable.*
☒	☐	For Bayesian analysis, information on the choice of priors and Markov chain Monte Carlo settings
☒	☐	For hierarchical and complex designs, identification of the appropriate level for tests and full reporting of outcomes
☐	☒	Estimates of effect sizes (e.g. Cohen's d, Pearson's r), indicating how they were calculated

Our web collection on statistics for biologists contains articles on many of the points above.

Software and code

Policy information about availability of computer code

Data collection	Not applicable, see fieldwork below
Data analysis	"WRS2" library of the R statistics program; "gbm" and "dismo" libraries of the R statistics program; "ncdf" library of the R statistics program. As well as different functions available in the "ggplot2", "dplyr", "tidyr", "raster" and "sp" libraries of the R statistics program.

For manuscripts utilizing custom algorithms or software that are central to the research but not yet described in published literature, software must be made available to editors and reviewers. We strongly encourage code deposition in a community repository (e.g. GitHub). See the Nature Portfolio guidelines for submitting code & software for further information.

Data

Policy information about availability of data

All manuscripts must include a data availability statement. This statement should provide the following information, where applicable:

- Accession codes, unique identifiers, or web links for publicly available datasets
- A description of any restrictions on data availability
- For clinical datasets or third party data, please ensure that the statement adheres to our policy

The datasets generated during and/or analyzed during the current study are available from the corresponding author upon reasonable request.

Research involving human participants, their data, or biological material

Policy information about studies with human participants or human data. See also policy information about sex, gender (identity/presentation), and sexual orientation and race, ethnicity and racism.

Reporting on sex and gender	Not applicable
Reporting on race, ethnicity, or other socially relevant groupings	Not applicable
Population characteristics	Not applicable
Recruitment	Not applicable
Ethics oversight	Not applicable

Note that full information on the approval of the study protocol must also be provided in the manuscript.

Field-specific reporting

Please select the one below that is the best fit for your research. If you are not sure, read the appropriate sections before making your selection.

☐ Life sciences ☐ Behavioural & social sciences ☒ Ecological, evolutionary & environmental sciences

For a reference copy of the document with all sections, see nature.com/documents/nr-reporting-summary-flat.pdf

Ecological, evolutionary & environmental sciences study design

All studies must disclose on these points even when the disclosure is negative.

Study description	We analysed remote sensing data and 266 in situ observations recorded during the seasonal “bleaching window”, August to December (Supplementary Fig. 1) at the Mesoamerican Reef (MAR), during 2015-2017. In the analysis, we describe first the temporal and spatial patterns of bleaching severity. Then, we analyse the association of these patterns with the 23 exposure and sensitivity metrics selected to test their capacity to predict coral bleaching severity.
Research sample	Scleractinian corals and the level of bleaching observed, we identified corals to species or genus level in the Mesoamerican Reef.
Sampling strategy	The selected sites had consistent information in other regional databases (e.g., AGRRA-Healthy Reefs, protected areas) and we prioritized areas based on the experience and feasibility of the surveys achieved by local experts. The monitoring was conducted by volunteers from various partner institutions of the Healthy Reef Initiative within Mexico, Belize, Guatemala, and Honduras.
Data collection	To assess bleaching severity in corals, the “bar-drop” method was employed to survey a minimum of 150 to 200 individual coral colonies using a 1m PVC bar with 5 marks every 25 cm. We haphazardly placed the bar across the reef after 3 - 4 kick cycles.
Timing and spatial scale	August to December (Supplementary Fig. 1) at the Mesoamerican Reef (MAR), during 2015-2017.
Data exclusions	None
Reproducibility	All original and raw data are stored as well as the R codes for the complete analysis of the manuscript. From the creation of the summary data to the statistical analysis.
Randomization	The reef areas to be evaluated were randomly selected, with the only restriction being that it was a coral reef zone, adding that all the corals identified in this work were randomly selected.
Blinding	Manipulative experiments were not conducted in this study. Blinding is not applicable.

Did the study involve field work? ☒ Yes ☐ No

Field work, collection and transport

Field conditions	Field conditions were determined by data collectors, and required low wind, low bottom turbidity, low waves, and conditions that would allow safe navigation in the ocean as well as optimal identification of the condition of the identified corals.
Location	69 sites in 2015; 104 sites in 2016; and 93 sites in 2017 in the Mesoamerican Reef, Atlantic Ocean (See Map Fig. 1 in Manuscript).

Access & import/export	All data collectors were responsible for acquiring the permits and licenses required to obtain the data. In addition, no manipulation or extraction of organisms was performed in this study.
Disturbance	The field work was carried out by professionals trained and qualified in scuba diving, taking special care not to disturb marine life and always trying to maintain adequate buoyancy.

Reporting for specific materials, systems and methods

We require information from authors about some types of materials, experimental systems and methods used in many studies. Here, indicate whether each material, system or method listed is relevant to your study. If you are not sure if a list item applies to your research, read the appropriate section before selecting a response.

Materials & experimental systems

n/a	Involved in the study	
☒	☐	Antibodies
☒	☐	Eukaryotic cell lines
☒	☐	Palaeontology and archaeology
☐	☒	Animals and other organisms
☒	☐	Clinical data
☒	☐	Dual use research of concern
☒	☐	Plants

Methods

n/a	Involved in the study	
☒	☐	ChIP-seq
☒	☐	Flow cytometry
☒	☐	MRI-based neuroimaging

Animals and other research organisms

Policy information about studies involving animals; ARRIVE guidelines recommended for reporting animal research, and Sex and Gender in Research

Laboratory animals	Not applicable
Wild animals	Scleractinian corals were observed in the natural habitat without removal or manipulation of live organisms.
Reporting on sex	Not applicable
Field-collected samples	Not applicable
Ethics oversight	Not applicable

Note that full information on the approval of the study protocol must also be provided in the manuscript.

Plants

Seed stocks	Not applicable
Novel plant genotypes	Not applicable
Authentication	Not applicable
